# MicroRNA based theranostics for brain cancer: basic principles

**DOI:** 10.1186/s13046-019-1180-5

**Published:** 2019-05-29

**Authors:** George E. D. Petrescu, Alexandru A. Sabo, Ligia I. Torsin, George A. Calin, Mihnea P. Dragomir

**Affiliations:** 1 Carol Davila University of Medicine and Pharmacy, Bucharest, Romania; 2 Bagdasar-Arseni Clinical Emergency Hospital, Department of Neurosurgery, Bucharest, Romania; 3 Marie Curie Emergency Clinical Hospital for Children, Bucharest, Romania; 4 Elias Clinical Emergency Hospital, Anaesthesiology and Critical Care Department, Bucharest, Romania; 5 Department of Experimental Therapeutics, The University of Texas MD Anderson Cancer Center, Houston, TX USA; 6 Center for RNA Interference and Non-Coding RNAs, The University of Texas MD Anderson Cancer Center, Houston, TX USA

**Keywords:** microRNA, miRNA based drugs, Antagomirs, Antisense oligonucleotides, miRNA masks, Small molecule miRNA inhibitors, miRNA mimics, Biomarkers, Glioma, Glioblastoma

## Abstract

**Background:**

Because of the complexity of the blood-brain barrier (BBB), brain tumors, especially the most common and aggressive primary malignant tumor type arising from the central nervous system (CNS), glioblastoma, remain an essential challenge regarding diagnostic and treatment. There are no approved circulating diagnostic or prognostic biomarkers, nor novel therapies like immune checkpoint inhibitors for glioblastoma, and chemotherapy brings only minimal survival benefits. The development of molecular biology led to the discovery of new potential diagnostic tools and therapeutic targets, offering the premise to detect patients at earlier stages and overcome the current poor prognosis.

**Main body:**

One potential diagnostic and therapeutic breakthrough might come from microRNAs (miRNAs). It is well-known that miRNAs play a role in the initiation and development of various types of cancer, including glioblastoma. The review aims to answer the following questions concerning the role of RNA theranostics for brain tumors: (1) which miRNAs are the best candidates to become early diagnostic and prognostic circulating biomarkers?; (2) how to deliver the therapeutic agents in the CNS to overcome the BBB?; (3) which are the best methods to restore/inhibit miRNAs?

**Conclusions:**

Because of the proven roles played by miRNAs in gliomagenesis and of their capacity to pass from the CNS tissue into the blood or cerebrospinal fluid (CSF), we propose miRNAs as ideal diagnostic and prognostic biomarkers. Moreover, recent advances in direct miRNA restoration (miRNA mimics) and miRNA inhibition therapy (antisense oligonucleotides, antagomirs, locked nucleic acid anti-miRNA, small molecule miRNA inhibitors) make miRNAs perfect candidates for entering clinical trials for glioblastoma treatment.

## Background

Brain and other central nervous system (CNS) tumors have an incidence of 29.4 per 100.000 persons in the adult population and 31.5% of the newly diagnosed tumors are malignant. [1]. Gliomas are tumors of the CNS arising from the glial cells. Glioblastoma (grade IV) is the most common primary malignant brain tumor (47.1%) and is characterized by a poor prognosis despite the available multimodal treatment (5.5% survival rate at 5 years) [1]. This can be explained through their heterogeneity, chemoresistance and infiltrative pattern that makes complete resection difficult. Low-grade gliomas (LGG, WHO grade I-II) have better overall survival (OS) of approximately 7 years, but ultimately, they progress to high grade gliomas (HGG, WHO grade III-IV) [2]. The current standard of care protocol for glioblastoma includes maximal safe resection of the newly diagnosed lesion followed by radiotherapy and chemotherapy with temozolomide (TMZ) [3]. Regardless of this, recurrence of glioblastoma can be seen after a median of 6.9 months [4]. Bevacizumab in addition to chemo- and radiotherapy increases the progression-free survival for newly-diagnosed cases, but further studies are necessary to verify its efficiency in improving OS [3]. Due to the fulminant clinical course that HGG usually have, the diagnosis is generally too late. Unfortunately, in clinical practice, there are no blood markers that would make the early diagnosis possible [5].

The development of molecular biology led to the discovery of new potential diagnostic tools and therapeutic targets, offering promise to overcome the current poor prognosis and diagnose patients in earlier stages. One potential therapy is based on microRNAs (miRNAs).

The majority of the human genome is transcribed into non-coding RNA (ncRNA), and only 2–3% of the genome encodes protein-genes [6]. The most studied types of ncRNAs are miRNAs. MiRNAs are a class of small ncRNAs, made of approximately 22 nucleotides [7], that are involved in gene-regulation at the post-transcriptional level by inducing mRNA degradation and translational repression. Additionally, it was shown that miRNAs have also more complex mechanisms of action: activating transcription, up regulating protein expression, interacting with RNA binding proteins, binding to Toll-like receptors and inhibiting nuclear or mitochondrial transcripts [8]. Mature miRNAs or precursor transcripts are well-known to be involved in the mechanisms of carcinogenesis [9–12] and are potential new therapeutic targets and biomarkers.

This review aims to answer the following questions regarding the role of RNA theranostics for brain tumors: (1) which miRNAs are the best candidates to become early diagnostic and prognostic circulating biomarkers?; (2) how to deliver the therapeutic agents in the CNS to overcome the blood-brain barrier?; (3) which are the best methods to restore/inhibit miRNAs?

## Deregulation of miRNAs in brain tumors

### Role of miRNA dysregulation in gliomagenesis

It is known that miRNAs play a role in the initiation and development of various types of cancer [13, 14]. In the past few years, the role of miRNAs in gliomagenesis has been intensely studied. They can have tumor suppressor properties or can act as oncogenes.

The dysregulation of the protein complex NF-kappaB promotes tumor growth and angiogenesis in glioblastoma [15, 16]. The tumor suppressive miR-31 that targets TNF receptor associated death domain (TRADD) and inhibits NF-kappaB activation is deleted in the majority of HGGs and therefore tumor proliferation is increased [17]. MiR-16 also downregulates the NF-kappaB1/MMP9 pathway and is less expressed in glioma samples [18]. The same study found that miR-16 could induce apoptosis by inhibiting the expression of B-cell lymphoma 2 (BCL2), as previously described in chronic lymphocytic lymphoma [18, 19]. BCL2 is an anti-apoptotic mitochondrial protein also involved in the early stages of glioma cells proliferation and progression to HGG [19–21]. One recent paper described that miR-184 could act as a tumor suppressor miRNA in gliomas by targeting TNF-α-induced protein 2 [22].

### The microenvironment and the immune cells

Gliomas are able to manipulate the cells from the surrounding microenvironment and promote cancerous cell migration, growth and immune evasion [23]. The aggressiveness of GBM is partially caused by the inability of the immune system to detetc its growth [24]. Microglia are resident macrophage of the CNS, that play a role in immune surveillance and host defence [25]. But the morphological phenotype of the microglia and their immune marker profile is strongly influenced by microenvironmental factors [26, 27]. Microglial cells and macrophages can turn to an M1 phenotype (or classically activated macrophages) or an M2 phenotype (or alternatively activated macrophages) [28]. Granulocyte-macrophage colony stimulating factor (GM-CSF), lipopolysaccharide (LPS), tumor necrosis factor-α (TNF-α) and interferon-γ (INF-γ) promote the transformation of microglial cells to M1 phenotype [28, 29]. Through secretion of cytotoxic factors and presentation of tumor antigen to T helper type 1 cells (Th1) cells, M1 cells display their role in antitumoral immunity. [30]. Furthermore, by activation of STAT1, M1 cells produce pro-inflammatory cytokines and increase T-cell-mediated cytolysis [30, 31].

MiR-155, a pro-inflammatory miRNA, was directly linked to the M1 phenotype [32]. Glioma cells produce IL-1 which strongly upregulates miR-155 in glial cells [33]. MiR-155 is upregulated by LPS, TNF-α and INF-γ and targets the anti-inflammatory protein suppressor of cytokine signalling 1 (SOCS-1) [34]. Thus, miR-155 leads to an increase of a series of inflammatory mediators such as the inducible nitric oxide synthase, IL- 6, and TNF-α [34]. In glioblastoma, miR-155 is an onco-miRNA that is highly expressed and its levels gradually enhance with the increase of tumor grade [35]. MiR-155 knockdown enhanced the effect of temozolomide through the induction of MAPK13 and MAPK14-mediated oxidative stress and apoptosis, representing a potential target for the treatment of glioma [35]. MiR-146 is also induced by IL-1 and is upregulated in gliomas, being a negative-regulator of astrocyte-mediated inflammation [36, 37].

The activation of M2 phenotype cells is due to the presence of cytokines such as IL-4, IL-10, IL-13 and transforming growth factor-β (TGF-β) [28, 38]. The M2 cells further produce immunosuppressive factors and activate STAT3 [28]. STAT3 is a transcription factor which decreases the expression of surface molecules for antigen presentation and increases the expression of IL-10, vascular endothelial growth factor (VEGF) and matrix metalloproteinase, further promoting angiogenesis, matrix remodelling and suppression of adaptive immunity [38, 39].

Even with the particular immunological characteristics of the CNS, the microenvironment can be used to support immunotherapeutic options for the treatment of brain tumors [40].

## MiRNAs and the blood-brain barrier

### Molecular anatomy of the blood-brain barrier

One key obstacle in developing new drugs for CNS disorders is the delivery of the therapeutic agents across the blood-brain barrier (BBB). BBB represents a complex structure that controls the passing of the nutrients and oxygen from the blood stream to the brain and prevents the accumulation of neurotoxins in the CNS. Dedicated endothelial cells connected through tight-junctions (TJ) line the brain capillaries and interact with adjacent supporting cells (astrocytes, pericytes, mast cells) forming the neuro-vascular unit [41]. The astrocytes control the permeability and preserve the integrity of the BBB [42]. They also create a link to the neurons by outlining the basal lamina of the microvessels through their endfeet [43]. Pericytes are essential for the development of the BBB during embryogenesis. They are embedded in the basal lamina and have a role in vesicle transport and formation of TJ [41, 44]. The complex interactions between the endothelial cells and surrounding cells promote the secretion of cytokines and subsequently disrupt the integrity of the BBB and allow passage of circulating immune cells and pathogenic agents [45].

BBB allows the passage of cationic or small lipid-soluble molecules with a molecular weight under 400 Da [46]. Transporters carry glucose and amino acids, while molecules with a higher molecular mass, i.e., insulin and transferrin, enter the BBB through receptor-mediated endocytosis [47]. The barrier between the blood and cerebrospinal fluid (CSF) is formed by the adapted epithelial (ependymal) cells of the choroid plexus linked through TJs and the arachnoid membrane which is also made of cells connected by TJs [48]. Circumventricular organs (CVOs), such as the pituitary gland and vascular organ of *lamina terminalis*, have a microvasculature characterized by high-permeability, allowing high molecular mass polypeptide hormones to exit the brain [49]. The CVOs-CSF barrier is made of ependymal cells, whereas tanycytes (modified ependymal cells) form the brain-CVOs barrier [45].

### MiRNAs altering the BBB

Numerous studies reported that miRNAs can modulate the permeability and integrity of the BBB, especially in pathological settings. Extracellular vesicles (EVs) containing miR-181c disrupt the BBB and promote brain metastasis from breast cancer by downregulating 3-phosphoinositide-dependent protein kinase 1 (PDPK1), and subsequently altering the actin filaments [50]. Overexpression of miR-210 alters the BBB by targeting junctional proteins (occludin and *β*-catenin) and aggravates cerebral edema in neonatal rats with hypoxic-ischemic brain lesions [51]. Aquaporin-11 (AQP11) is a membrane protein located in the endothelial cells of the brain capillaries and the epithelial cells of the choroid plexus [52]. The BBB of AQP-11 deficient mice has no structural or functional changes [52]. However, a recent paper found that miRNA-27a-3p mimic targets the up-regulated AQP11 and has a protective effect on the integrity of the BBB in rats with intracerebral hemorrhage (ICH) [53]. MiR-98 and let-7 decrease the permeability of the BBB under neuroinflammatory setting by lowering the expression of cytokines and the adhesion of leukocytes [54]. TNF-*α* alters the TJs and therefore increases the permeability of the BBB [55]. TNF-*α* upregulates miR-501-3p in the white matter of mice with cerebral hypoperfusion which leads to an inhibition of zonula occludens-1 (ZO-1) protein and lowers the transendothelial electric resistance [56]. MiR-125a-5p overexpression in endothelial cells leads to the formation of stronger junctional complexes between ZO-1 and vascular endothelial cadherin (VE-cadherin) [57].

### How do miRNAs overcome the BBB?

Current evidence suggests that the BBB is not blocking the passage of miRNAs between CSF and blood, but they have a more diluted concentration in blood than CSF [58]. It is known that in pathological states miRNAs can pass from the brain tissue into the blood stream through the BBB, making them potential biomarkers for CNS diseases [59]. On the other hand, very little data exists regarding the passage of miRNAs from blood into the brain tissue. It is known that siRNAs, which have a molecular mass of 14 kDa, similar to the miRNAs, cannot diffuse through the BBB [60].

## MiRNAs as potential therapeutic tools

**Fig. 1:**
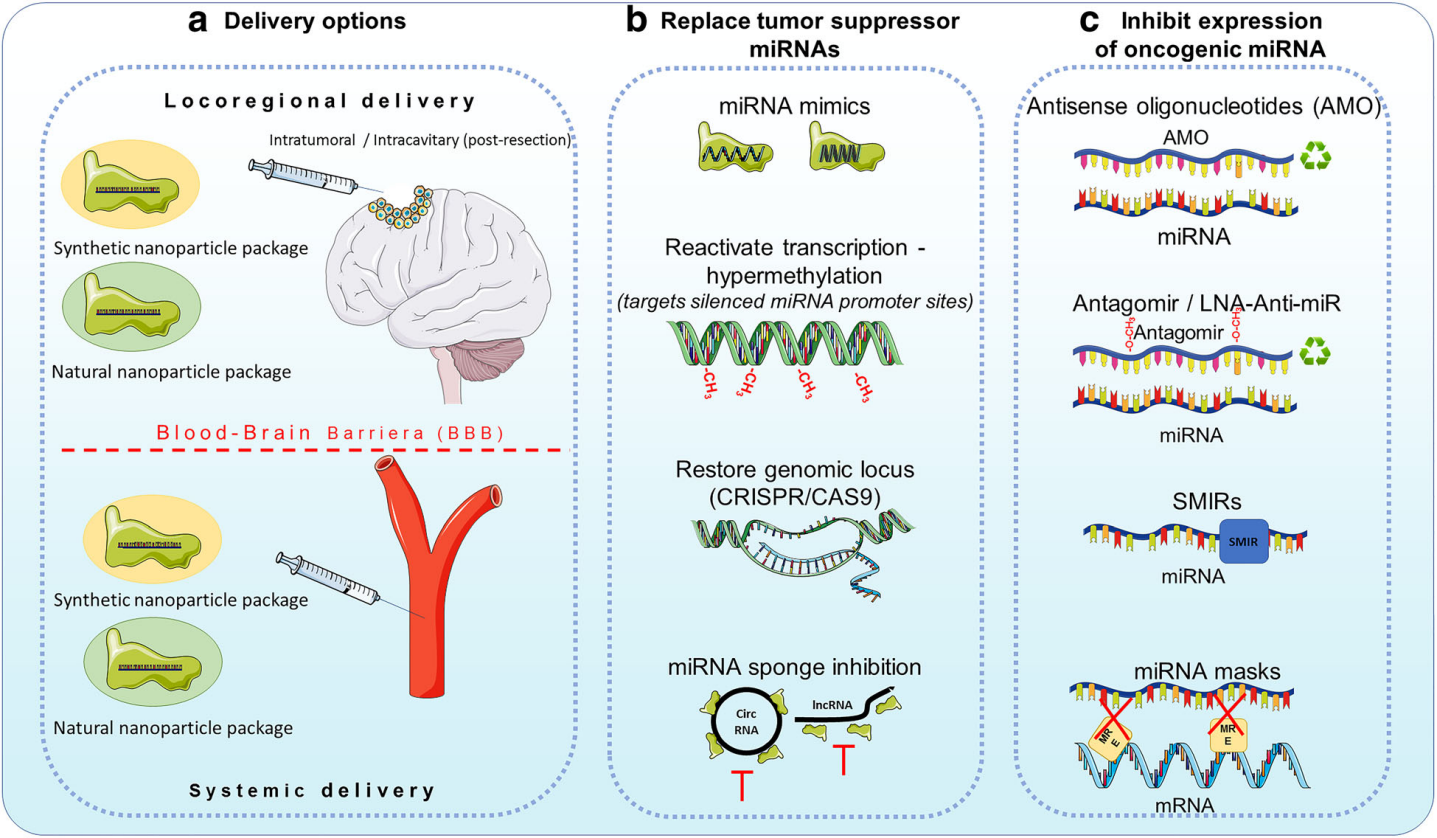
MiRNA therapy for glioblastoma. MiRNA therapy can be classified into miRNA restoration therapy (i.e. restoring tumor suppressor miRNAs) and miRNA inhibition therapy (inhibiting oncomiRs). **a** The delivery of this potential therapy is hindered by the selective structure of the blood brain barrier (BBB). We can envision two possible delivery methods – locoregional (post-surgery) and systemic. Locoregional is invasive but the BBB is directly by-passed, the systemic delivery on the other hand is less invasive and can be repeated multiple times. The most suitable carriers of this therapy are nanoparticles, which can be synthetic or natural, by offering the advantage of a higher half-time for the therapeutic agent, at a lower dose and with fewer side effects. **b** The methods to achieve miRNA restoration therapies can be direct: delivery of miRNA mimics – single/double strand synthetic RNA molecules that mimic the function of endogenous miRNAs or indirect: reactivation of transcription by using hypomethilating drugs (Decitabine or 5-azacytidine); restoring the genomic locus of a miRNA using Crispr/CAS9 or vectors expressing the missing miRNA or inhibiting ceRNA molecules that sponge anti-tumorigenic miRNAs. **c** The inhibition of oncomiRs can be realized by AMOs (antisense oligonucleotides) that covalently bind mature miRNAs and induce their degradation; antagomirs or LNA anti-miRs which are chemically modified antisense RNA molecules, that have a higher stability and a lower degradation level compared to AMOs; small molecule miRNA inhibitors (SMIRs) which block the function of specific miRNAs by structure-based binding to the precursor or mature form of miRNA; and miRNA masks which block the miRNA response elements (MREs) on mRNAs so that miRNAs cannot achieve their inhibitory function

In order to overcome this limitation, several delivery methods have been developed. There are two main delivery routes that can be used, locoregional (that is used to by-pass the BBB) or systemic (that needs to penetrate the BBB) and two types of packaging nanoparticles, natural or synthetic. Locoregionally, nanoparticles can be stereotaxically administered *directly into the tumor*, or can be delivered in the *tumor resection cavit*y through biodegradable wafers or convection-enhanced delivery (CED) [61]. Other methods include *intrathecal* delivery directly into the CSF or placement of an Ommaya reservoir (*intraventricular* catheter connected to a reservoir placed under the scalp that is used for the delivery of drugs) [61, 62]. For systemic delivery, natural (exosomes), as well as synthetic particles (liposomes, gold nanoparticles) have been used (Fig. 1a) [63–66]. The development of tumors in the CNS also leads to the disruption of the BBB, making it easier for molecules to pass the BBB, but given the characteristics of the tumor vessels, the molecules also have a higher clearance [67].

Regarding the load of the nanoparticles, two fundamental strategies can be envisioned: (1) restoring the downregulated tumor suppressor miRNAs or (2) inhibiting the overexpressed oncomiRs.

Restoring the downregulated tumor suppressor miRNAs can be achieved with miRNA mimics, which are synthetic double strand RNA molecules with identical sequence as natural miRNAs that are able to integrate into the RNA induce silencing complex (RISC) and perform the anti-tumorigenic function of the missing miRNA. It was also proven that single strand RNA mimetic therapy is achievable in the brain tissue. Yu et al. injected single strand RNA molecules directly into the brain of mice and inhibited mutant Huntington proteins [68]. Recently, it was shown that in vivo administration of miR-138, an inhibitor of both CTLA-4 and PD-1, induces tumor regression and prolongs the survival of immune-competent mice, but not of immune incompetent mice [69]. It seems that miR-138 is an ideal immune therapy for gliomas.

The levels of a tumor suppressor miRNA can be restored also indirectly, by reactivating the transcription (targeting hypermethylation of silenced miRNA promoter sites [70]; restoring a deleted genomic locus at the DNA level (CRISPR/Cas9) or by inhibiting possible miRNA sponges (long non-coding RNAs (lncRNAs) or circular RNAs (circRNAs)) (Fig. 1b) which seem to be more abundant in the brain, building complex coregulatory networks [71].

Anti-miRNA therapy aims to inhibit the expression of oncogenic miRNAs which are overexpressed in the tumor. Multiple mechanisms had emerged recently, that could be translated into clinical practice. MiRNA inhibition can be achieved by antisense oligonucleotides (AMOs), miRNA masks, antagomirs, locked nucleic acid (LNA) anti-miRNAs, small molecular miRNA inhibitors (SMIRs) and miRNA sponges.

AMOs are single RNA strands, that have a length similar to miRNAs (approximately 20 nt) and that can complementary and specifically bind to a mature miRNA, leading to its inhibition [72, 73]. AMOs form together with their target miRNAs RNA duplexes which lead to the degradation of miRNAs by RNAse H. In order to function in vivo, AMOs require chemical modifications like 2′-O-methoxyethyl and phosphorothioate. Oh et al. showed that by administering anti-miR-21 antisense oligodeoxynucleotide carrier by R3V6 peptide which has amphiphilic properties, directly in the glioblastoma of a xenograft animal model, the apoptosis of tumor cells was restored and consequently tumor growth was blocked [74].

Antagomirs are single strand RNA molecules, containing 2′-methoxy groups and phosphorothioates, and cholesterol conjugated in order to hinder degradation, perfectly complementary to mature miRNAs. Antagomirs form RNA duplexes with their miRNA target, leading to the degradation of the miRNA and the recycling of the antagomir [75]. When administered in murine models harboring U87 glioblastoma tumors, antagomir-27a, the proliferation and invasiveness were reduced by upregulating the tumor suppressor FOXO3a [76].

LNA anti-miRs are AMOs in which the 2′-O and 4′-C atoms of the ribose ring are connected through a methylene bridge, decreasing the flexibility of the ring and inducing a rigid conformation [77]. These chemical changes confer increased nuclease resistance and increased binding affinity of LNA anti-miRs to their target miRNAs [78]. Systemically delivery of anti-miR-21-LNA coupled with multivalent folate (FA) conjugated three-way-junction-based RNA nanoparticles (RNP) (FA-3WJ-LNA-miR21 RNP) in an orthotopic glioblastoma xenograft mouse model promoted the apoptosis of glioblastoma cells [79]. Other study showed that by administering LNA-anti-miR21 and neural precursor cells (NPC) that deliver a secreting type of tumor necrosis factor–related apoptosis inducing ligand (S-TRAIL) in murine glioblastoma models, a synergistic effect is obtained leading to a reduced tumor volume [80].

SMIRs are small molecule chemical compounds that bind precursor or mature miRNAs and prevent their biogenesis, maturation or function [81]. AC1MMYR2 blocks the maturation of pre-miR21, leading to tumor suppression in orthotopic mouse models [82].

The arsenal of anti-miRNA therapy is completed by miRNA sponges. This strategy is based on the role of other ncRNAs (i.e. lncRNAs and especially circRNAs) to bind and inhibit the function of miRNAs. MiRNA sponges can be specifically synthesized with multiple miRNA binding site, and loaded into tumor cells, so that a potent inhibition of oncogenic miRNAs can be reached. This therapeutic method is appealing because recent data show that circRNAs are abundant in the brain and function as natural sponges [83, 84]. Cell lines and orthotopic glioblastoma mice models infected with miR-23b sponge expressing lentivirus had decreased angiogenic, infiltration and migration properties by downregulating MMP2, MMP9, VEGF, HIF-1α, β-catenin, and ZEB1 and upregulating VHL and E-cadherin [85]. Indirect inhibition of miRNAs is realized by miRNA masks. MiRNA masks bind to the miRNA binding site on the mRNA, called miRNA response element (MRE), and protect the mRNA from miRNA inhibition [86] leading to an up-regulation of the suppressed oncomiR targets.

Nadaradjane et al. demonstrated that miRNAs can also be used to decrease the chemoresistance of glioblastoma cells [87]. By administering in glioblastoma mice models miR-370-3p and TMZ the tumor volume reduced by two-fold when compared to TMZ alone. Also, orthotopic xenografts of P-GBM2 cells with miR-198 overexpressed, showed a significant decrease of chemoresistance to TMZ and reduced tumor growth [88]. Chen et al. showed that in GBM xenografts treated with miR-181b the tumor growth was suppressed and the sensitivity to TMZ was increased through the downregulation of EFGR [89].

Intravenously delivery of miR-142-3p lead to an increased survival of mice bearing GL261 tumor cells by inducing the apoptosis of M2 immunosuppressive macrophages [90]. Finally, miRNA therapy can be combined with oncolytic viral treatments. Semliki Forest virus-4 (SFV-4) has oncolytic properties. Systemically delivery of engineered SFV-4miRT (containing target sequences for miR-124, miR-125 and miR-134 to reduce its neurovirulence) increased the survival of glioma and neuroblastoma mice models [91].

When administered intravenously in murine glioma models, miR-124 led to an inhibition of glioma growth. The same effect was observed when miR-124-transfected T-cell were adoptively transferred into tumor-bearing mice. MiR-124 inhibited STAT3 pathway and reversed glioma stem cells mediated immune suppression of T-cell proliferation and induction of Forkhead box P3 regulatory T cells [92].

More recently, two papers explored the therapeutic effect of manipulating more than one miRNA. Bhaskaran et al. demonstrated that combined administration of multiple miRNAs, miR-124, miR-128, miR-137, which inhibit multiple oncogenes, and chemotherapy, led to an increased survival in intracranial GBM murine models. Also, interestingly, in vivo data showed that, the cells overexpressing these miRNAs deliver the miRNA cluster to nearby cells via EVs and subsequently promote a widespread antitumoral effect [93].

By running an in silico analysis based on differentially expressed miRNAs in GBM and their target genes, Xiong et al. identified three new potential miRNA-based agents for GBM therapy (gefitinib, exemestane and W-13) [94]. Using this approaches one might resolve the heterogeneity problem that arises in GBM.

## MiRNAs as potential diagnostic tools

A biomarker is a biological indicator, that can be objectively measured, which reflects the risk or presence of a disease [95]. The utility of biomarkers in managing brain tumors has grown in importance over the past decades, some being already used in daily medical practice, e.g. the methylation of the promoter of the gene for O^6^-methylguanine-DNA methyltransferase (MGMT). In the latest WHO classification of CNS tumors, molecular characteristics are taken into account to define the diagnosis [96]. One of the extensively studied biomarkers are miRNAs, and although they are not currently used in clinical practice; advances in this field show that their utility in the oncologic diagnostic process may be crucial, and could replace specific steps in current diagnostic practices. For example, replacing a traditional tissue biopsy with a so called “liquid biopsy” would spare the patient and the doctor a diagnostic surgical intervention. Also, given the heterogeneity of gliomas, using only a small tissue sample obtained from surgery or a biopsy could lead to an undergrading, like it was demonstrated for Isocitrate Dehydrogenase (IDH) wild-type gliomas [97]. More than that, biomarkers could indicate patient prognosis, guide the treatment, and be used as a screening tool in the follow-up process. But in order to do that, they need to be highly specific, standardized and reliable.

In CNS disorders, the liquid biopsy can be performed by studying either blood or CSF samples. While obtaining a blood sample is less invasive, using CSF can be more reliable since it is in close contact with CNS structures and has a higher miRNA concentration [58, 98].

Regarding blood derived products (Table 1), one of the most studied single miRNA is miR-21. A 2015 meta-analysis pinpointed this miRNA to be the most powerful single miRNA in brain cancer diagnostics [99]. In one study, it has been shown that, alone, miR-21, can differentiate between glioma and healthy controls with sufficient sensitivity and specificity. Still, in the same study, it was not possible to distinguish between glioma and other brain tumors (meningiomas or pituitary tumors) [100]. Two other studies include mir-21 in a three-miRNA panel, D’Urso et al. propose a diagnostic tree, by adding mir-15b to differentiate between glioma and other conditions (including neurologic conditions, brain metastases and Primary Central Nervous System Lymphoma (PCNSL)), and mir-16 to differentiate between different grades of glioma [101]. Besides miR-21, Santangelo et al. add miR-222 and miR-124-3p to distinguish between glioma grades and healthy controls and report post-surgical normalization of miRNA serum levels, outlining their potential use in monitoring disease recurrence [102].

**Table 1:** MiRNAs from blood derived products (Serum/Plasma/Blood cells) as brain tumor biomarkers

miRNA	↑/↓	Study, Year, Ref.	Biological fluid and Analysis method	No. of pts.	Significance	Area under the Curve (AUC) Sensitivity (SS) Specificity (SP)
miR-21	↑	Wang, 2012 [100]	Plasma	30 Glioma	Distinguishes between GBM and healthy controls	Glioma vs. healthy controls
			qRT-PCR	(10 Gr II)	Expression levels cannot distinguish between glioma grades	AUC = 0.9300 (95% CI: 0.7940–1.066)
				(10 Gr III)	Cannot distinguish between glioma and other brain tumors	SS = 90.0%
				(10 Gr IV)		SP = 100%
				10 Meningioma		
				10 Hyphophysoma		
				10 Healthy controls		
miR-29	↓	Wu, 2015 [106]	Serum	83 Glioma	Distinguishes HGG^a^ from healthy controls	AUC = 0.81 (95% CI, 0.73–0.89).
			qRT-PCR	(36 Gr I-II)	Not a brain cancer specific marker	
				(47 Gr III-IV)		
				69 Healthy controls		
miR-21	↑	D’Urso, 2015 [101]	Blood	30 Glioma	Combined diagnostic tree using miR-15b and miR-21 can distinguish glioma from other conditions	Combined miR-15b and mir21
miR-15b	↑		Microarray	(8 Gr II)		SS = 90%
miR-16	↓		qRT-PCR	(6 Gr III)	Mir-16 levels could distinguish between grades of Glioma (lowest expression in GBM)	SP = 100%
				(16 Gr IV)		miR-16 to distinguish between Gr IV and II/III
				30 Various neurological		AUC = 0.98
				disorders		SS = 0.98%
				36 PCNSL^c^		SP = 99%
				16 Secondary brain		
				metastases		
miR-21	↑	Santangelo, 2018	Serum	100 Glioma	Higher serum levels of 3 miRNA panel in GBM and HGG compared to LGG^b^ and healthy controls	Cumulative 3 miRNA panel; GBM vs healthy:
miR-222	↑	[102]	Exosomes	(2 Gr I)	Cumulative 3 miRNA panel distinguishes between GBM, HGG, LGG and healthy controls	AUC = 0.87 (95% CI 0.7885–0.9524, *p* < 0.0001)
miR-124-3p	↑		qRT-PCR	(13 Gr II)	High serum levels return to normal postoperatively	SS = 84%
				(16 Gr III)		SP = 77%
				(69 Gr IV)		*For HGG, LGG, Metastases vs *healthy control, see original article*
				11 Brain metastases		
				30 Healthy controls		
miR-203	↓	Chen, 2017 [107]	Serum	70 GBM^d^	Distinguishes between GBM and LGG, GBM and healthy controls	GBM vs LGG
			qRT-PCR	30 LGG	Lower serum level correlated with larger tumor size, lower KPS^e^ score, lower OS^f^ and lower PFS^g^	AUC = 0.814
				30 Healthy controls		GBM vs healthy controls
						AUC = 0.862
miR-137	↓	Li, 2016 [108]	Serum	64 glioma	Downregulated levels in glioma compared to controls	NA
			qRT-PCR	(35 Gr I/II)	Further downregulation in HGG	
				(29 Gr III/IV) 64 Controls	Low levels associated with lower OS of glioma patients	
miR-185	↓	Tang, 2015 [103]	Serum	66 Glioma	Downregulation of mir-185 specifically associated with glioma patients compared to oncologic non-glioma patients	NA
			qRT-PCR	(23-Gr I + II)	Lower serum mir-185 levels in Grade III-IV glioma compared to Grade I-II	
				(43-Gr III + IV)	Lower mir-185 levels correlated with lower OS	
				11 Pituitary adenoma	Up-regulation of mir-185 levels after chemoradiation	
				32 Meningioma		
				14 Acoustic neuroma		
miR-210	↑	Lai, 2015 [109]	Serum	136 Glioma	Upregulation of mir-210 can distinguish glioma from healthy controls	Overall glioma (Gr I-IV) vs healthy controls
			qRT-PCR	(13 Gr I)	miR-210 levels associated with tumor grade	AUC value of 0.927 (95% CI1/40.889–0.964)
				(35 Gr II)	*(Upregulation trend in glioma Gr I-II* vs *healthy controls; Statistical significant upregulation in Gr III-IV* vs *healthy controls)*	NPV = 72.5%
				(46 Gr III)	High mir-210 levels associated with lower OS	PPV = 91.3%
				(32 Gr IV)		SS = 91.27%
				50 Healthy controls		SP = 72.50%
miR-205	↓	Yue, 2016 [104]	Serum	64 Glioma	Significant downregulation of mir-205 in all grades glioma compared to healthy controls	Overall glioma (Gr I-IV) vs healthy controls
			qRT-PCR	(7 Gr I)	Stepwise decrease in serum mir-205 levels with ascending pathological grades	AUC = 0.935
				(9 Gr II)	Significantly lower mir-205 levels in glioma versus other brain-tumors	PPV = 96.4%,
				(21 Gr III)	Downregulation of mir-205 correlated with KPS score and OS	NPV = 65.8%,
				(27 Gr IV)		SS = 86.3%,
				45 Healthy controls		SP = 92.2%,
				8 Meningioma		
				6 PCNSL		
				5 Pituitary adenoma		
miR-221/222 Family	↑	Zhang, 2016 [110]	Serum	50 Glioma	Distinguishes glioma from healthy controls	miR-221:
			qRT-PCR	51 Healthy controls		AUC = 0.84 (95% CI: 0.74–0.93)
						miR-222:
						AUC = 0.92 (95% CI 0.87–0.94)
miR-301a	↑	Lan, 2018 [105]	Serum exosomes	60 Glioma	Higher levels in glioma vs controls	NA
			qRT-PCR	43 Heallthy controls	Higher levels in glioma vs other types of cancers	
				9 Meningioma	Higher levels correlated with ascending pathological grades and lower KPS	
				7 PCNSL	Levels decrease postoperatively	
				10 Pituitary adenoma	Secondary increase may reflect local recurrence	
					Serum levels in HGG are independently associated with longer OS	
miR-397a	↓	Huang, 2017 [113]	Serum	100 Glioma	Distinguishes glioma from healthy controls	miR-376a: AUC = 0.872; SS = 81.0%; SP = 82.0%
miR-397b	↓					
miR-397c	↓					
			qRT-PCR	(10 Gr I)	Decreased levels associated with advanced WHO grade and low KPS	miR-376b: AUC = 0.890; SS = 82.0% SP = 78.0%;
				(20 Gr II)	Higher miRNA levels associated with better OS	miR-376c: AUC = 0.837; SS = 90.0%; SP = 70.0%
				(30 Gr III)		
				(40 Gr IV)		
				150 Healthy controls		
miR-122	↓	Tang, 2017 [114]	Plasma	74 Glioma	Distinguishes between glioma and healthy controls	AUC = 0.939
			qRT-PCR	(14 Gr I)	Further downregulation of serum levels in higher grade gliomas	SS = 91.9%
				(17 Gr II)	The miRNA level is an independent prognostic factor for OS	SP = 81.1%
				(20 Gr III)		
				(23 Gr IV)		
				74 Healthy controls		
miR-125b	↓	Regazzo, 2016 [112]	Serum	22 Glioma	Distinguishes between GBM and lower grade (II/III) gliomas	miR-125b: GBM vs lower grade glioma, AUC = 0.75 (95%
miR-497	↓					
			qRT-PCR	(12 Gr II/III)		CI = 0.533–0.967)
				(10 Gr IV)		miR-497: GBM vs lower grade glioma, AUC = 0.87 (95%
				8 Meningioma		confidence interval (CI) = 0.712–1
				15 Healthy controls		
miR-125b	↓	Wei, 2016 [111]	Serum	33 Glioma	Distinguishes between glioma and healthy controls	Glioma vs healthy controls
			qRT-PCR	(11 Gr I)		AUC = 0.839 (95% CI: 0.743–0.935)
				(11 Gr II)		
				(11 Gr III/IV)		
				33 Healthy controls		
miR-182	↑	Xiao, 2016 [115]	Serum	112 Glioma	Distinguishes between glioma and healthy controls	Glioma vs healthy controls
			qRT-PCR	(18 Gr I)	The expression levels associated with KPS score and WHO grade and correlated with lower OS and DFS,	AUC = 0.778
				(23 Gr II)	The level is independent prognostic factor for OS	SS = 58.5%
				(32 Gr II)		SP = 85.2%
				(39 Gr IV)		
				54 Healthy controls		
miR-128	↓	Sun, 2015 [121]	Serum	151 Glioma	Distinguishes between glioma and healthy controls and meningioma	Glioma vs healthy controls
			qRT-PCR	(24 Gr I)	Distinguishes Gr II-IV from Gr I	AUC = 0.9095
				(23 Gr II)	Levels elevated after surgery and correlate with the pathological grade and KPS	Glioma vs Meningioma
				(43 Gr III)		AUC = 0.8283
				(61 Gr IV)		Glioma II-IV vs I
				59 Post-op glioma		AUC = 0.7362
				52 Meningioma		
				53 Healthy controls		
				30 Glioma		
miR-128	↓	Wang, 2012 [100]	Plasma	(10 Gr II)	Can distinguish between GBM and healthy controls	AUC (miR-128 or miR-342-3p) = 1.000 (95% CI: 1.000–1.000)
miR-342-3p	↓		qRT-PCR	(10 Gr III)	Decreased levels correlated with glioma grade	SS = 90.0%
				(10 Gr IV)	Significant upregulation after operation and chemoradiation	SP = 100%
				10 meningioma		
				10 Hyphophysoma		
				10 Healthy controls		
miR-128	↑	Roth, 2011 [120]	Blood cells	20 Glioblastoma	Distinguishes between GBM and healthy controls	GBM vs healthy controls
miR-342-3p	↓		Microarray	20 Healthy Controls	*Authors apply statistic learning techniques (SVM = Support vector machines) and compute the diagnostic accuracy of miRNA profiles; a 180 miRNA signature calculated to have the highest diagnostic accuracy in distinguishing GBM from healthy controls.*	miR-128:
			qRT-PCR			AUC = 0.828
						miR-342-3p
						AUC = 0.18
						180 miRNA signature:
						SS = 83%
						SP = 79%
RNU6–1	↑	Manterola, 2014	Serum exosomes	Initial screening	3 small non coding RNAs can distinguish between GBM and healthy controls	RNU6–1
miR-320	↑	[119]	Low density array	25 Glioblastoma	(machine learning algorithm)	AUC = 0.852 (95% CI, 0.74–0.96)
miR-574-3p	↑		qRT-PCR	25 Healthy controls		SS = 73%; SP = 70%
				Confirmation	*Results from initial screening not confirmed is the second study group, only RNU6–1 was found significantly up-regulated!*	miR-320
				50 Glioblastoma		AUC = 0.720
				30 Healthy controls		(95% CI, 0.56–0.87)
						SS = 65%; SP = 65%
						miR-574-3p
						AUC = 0.738 (95% CI, 0.58–0.89)
						SS = 59%; SP = 59%
						3 sncRNA signature:
						AUC = 0.926 (95%[CI], 0.84–1)
						SS = 87%; SP = 86%
miR-454-3p	↑	Shao, 2015 [117]	Plasma	70 Glioma	Distinguishes between glioma and healthy controls	Glioma vs healthy controls
			qRT-PCR	(8 Gr I)	Higher levels in higher WHO grades and the levels decrease significantly postoperatively	AUC = 0.9063 [95% (CI): 0.8487–0.9639)]
				(15 Gr II)	Weak correlation between high levels and OS	SS = 99.05%
				(25 Gr III)		SP = 82.86%
				(22 Gr IV)		
				70 Healthy controls		
miR-451a	↓	Zhao, 2016 [116]	Serum	118 Glioma	Distinguishes between glioma and healthy controls	Glioma vs healthy controls
			qRT-PCR	(27 Gr I)	Levels return to almost healthy control expression 7 days after surgery	AUC = 0.816
				(33 Gr II)	The expression level downregulation correlates with WHO grade and KPS	SS = 81.4%
				(33 Gr III)		SP = 79.7%
				(25 Gr IV)		
				84 Healthy controls		
miR-15b-5p	↓	Yang, 2013 [122]	Serum	148 Glioma	Significantly decreased in glioma (Gr I-IV) compared to healthy controls	SS = 88.00%
miR-23a	↓					
miR-133a	↓					
miR-150*	↓					
miR-197	↓		Solexa sequencing	(15 Gr I)	Malignant astrocytoma prediction	SP = 97.87%
miR- 497	↓					
miR-548b-5p	↓		qRT-PCR	(55 Gr II)	Significant postoperative upregulation of aforementioned miRNAs	
				(45 Gr III)		
				(33 Gr IV)		
				11 Astrogliosis		
				80 Healthy controls		
miR-15b-5p	↑	Zhi, 2015 [123]	Serum	90 Glioma	Combined 9 miRNA panel distinguishes glioma from healthy controls	AUC = 0.9722 (95% CI, 0.9501–0.9942)
miR-16-5p	↑					
miR-19a-3p	↑					
miR-19b-3p	↑					
miR-20a-5p	↑		TaqMan	(28 Gr II)	Levels decrease postoperatively	SS = 93.3%
miR-106a-5p	↑					
miR-130a-3p	↑		Low density Array	(38 Gr III)	*miR-20a-5p, miR-106a-5p, and miR-181b-5p associated with advanced glioma stages*	SP = 94.5%
miR-181b-5p	↑					
miR-208a-3p	↑					
			qRT-PCR	(24 Gr IV)	*miR-19a-3p, miR-106a-5p, and miR-181b-5p significantly associated lower OS.*	
				110 Healthy controls		
miR-17	↑	Xu, 2017 [118]	Serum	47 Glioma	Distinguishes between glioma and healthy controls	miR-17; AUC = 0.787 [95% (CI): 0.690–0.865)] SS = 89.3%; SP = 55.3% miR-130a AUC = 0.720 [95% (CI): 0.617–0.807)] SS = 70%; SP = 65.2% miR-10b AUC = 0.721 [95% (CI): 0.619–0.808)] SS = 44.6%; SP = 93.6% miR-Score (all three miRNA) AUC = 0.872 [95% (CI): 0.787–0.932)] SS = 72.3%; SP = 85.1%
miR-130a	↑		qRT-PCR	(16 Gr I-II)	Higher serum levels in HGG compared to LGG	
miR-10b	↑			(31 Gr III-IV)		
				45 Healthy controls		
miR-93	↑	Goze, 2018 [124]	Whole blood	15 DLGG^h^	3 miRNA signature tree distinguishes DLGG from healthy controls	miRNA-93; AUC = 0.83556
miR-590-3p	↑					
miR-454	↑		TaqMan OpenArray RT-qPCR platform	15 Healthy controls		miRNA-590-3p; AUC = 0.8133
						miRNA-454; AUC = 0.75111

^1^HGG High grade glioma, ^2^LGG Low-grade glioma, ^3^PCNSL Primary central nervous system lymphoma, ^4^GBM Glioblastoma, ^5^KPS Karnofsky Performance Scale, ^6^OS Overall Survival, ^7^PFS Progression free survival, ^8^DLGGdiffuse large grade glioma

Some studies compared glioma patients to patients suffering from other brain cancers and healthy controls, miR-185 has been shown to be significantly decreased in glioma, compared to other brain cancers. Also, the serum levels of the same miRNA have been linked to worse prognosis [103]. Similarly, miR-205 has been shown to differentiate between all-grades glioma and healthy controls, and to be significantly decreased in glioma compared to meningioma, PCNSL and pituitary adenoma. More than that, the levels are linked to lower Karnofsky Performance Scale (KPS) score and worse OS [104]. Likewise, levels of miR-301 have been also screened in other brain cancers – meningioma, PCNSL and pituitary adenoma and glioma. The levels of miR-301 are shown to be significantly dysregulated in glioma. Also, serum levels of miR-301 were related to KPS score and normalize postoperatively, suggesting the possible use of this miRNA in recurrence screening [105].

Other studies compare glioma patients with healthy controls only, and focus on different single miRNA dysregulation: miR-29 can be used to distinguish between high grade glioma and healthy controls [106]; miR-203 helps to differentiate glioblastoma from low-grade glioma and healthy controls and is linked with lower KPS and OS [107]; miR-137 is stepwise down-regulated in higher glioma grades and predicts lower OS [108]; miR-210 can be used to distinguish between all grade gliomas and healthy controls [109]; miR-221/222 family might differentiate glioma from healthy controls (grades not specified in this study) [110]; mir-125 alone [111] or together with miR-497 [112] are able to distinguish between glioma grades and healthy controls; miR-397a, b, c [113] miR-122 [114], and miR-182 [115] can distinguish glioma from healthy controls and are related to worse overall survival; miR-451a [116] and miR-454-3p [117] differentiate glioma from healthy controls, and their serum levels return to normal after surgery. Xu et al. propose a three miRNA signature (miR-17, miR-130a, miR-10b) to differentiate between glioma and healthy controls [118]. Likewise, Manterola also suggests a three small RNA signature including two miRNAs (miR-320, miR-574-3p) and RNU6–1**,** that can differentiate between GBM and healthy controls, but only the latter withstands their validation study and is significantly upregulated [119].

Two miRNAs – miR-128 and miR-342-3p have been both reported by 2 different studies to be useful in the differentiation of glioblastoma from healthy controls. Mir-128 has been reported to be upregulated in one study, while being downregulated in the other, a possible explanation for this fact being the different biofluids used for miRNA analysis, one using plasma, the other whole blood cells [100, 120]. One of the studies also reported the post-surgical and post-chemoradiation miRNA upregulation [100]. Interestingly, a third study focusing on mir-128**,** reports its ability to differentiate between glioma and healthy controls. Also, it mentions a good ability to differentiate Grade I from Grade II-IV. Besides that, its serum level elevation after surgery is linked to a lower KPS score [121].

Other studies use multiple miRNA signatures as biomarkers. Yang et al. propose a highly accurate seven miRNA panel [122]; Zhi et al. a nine miRNA panel [123], both studies being able to distinguish glioma from healthy controls, while showing postoperative normalization of serum levels.

While most of the studies focus on high grade glioma, Goze et al. propose three miRNAs signature (miR-93, miR-590-3p, and miR-454) to differentiate diffuse LGG from healthy controls [124].

Regarding CSF miRNA analysis (Table 2), miR-21 upregulation has been reported by several studies to differentiate between glioblastoma and healthy controls [125–127]. Still, miR-21 expression levels in CSF could not distinguish between CNS metastases and PCNSL [125, 127]. Likewise, miR-10b is not normally found in healthy brain tissue (ergo, not in CSF), its presence indicating a malignant brain process. Despite this, miR-10b is not able to differentiate glioblastoma from brain metastases [127]. Likewise, miR-200 is not normally present in CSF of healthy individuals but is overexpressed in both glioma and brain metastases. The levels of expression are significantly higher in the metastases, therefore, making it a promising tool in differentiating glioblastoma from metastases [127]. Similarly, miR-15b CSF levels have been reported to be markedly elevated in glioblastoma compared to PNCSL and metastases. Therefore, the authors propose an accurate diagnostic tree using miR-15b and miR-21 [125]. Two other studies focused on CSF miRNA signatures in glioblastoma. Akers et al. propose a nine-miRNA panel after testing CSF tapped from two distinct locations – cisternal and lumbar, proving a relatively high sensitivity in the first (80%), and a relatively low in the latter (28%), in distinguishing glioblastoma from healthy controls. However, the utility of cisternal CSF diagnostics is limited to selected patients with an implanted ventriculo-peritoneal shunt or an Ommaya reservoir [128]. Interestingly, Drusco et al. analyzed a set of primary and secondary brain tumors and proposed a diagnosis diagram based on this five miRNA panel to differentiate between types of brain tumors [129].

**Table 2:** MiRNAs from CSF as brain tumor biomarkers

miRNA	↑/↓	Study, Year, Ref.	Biological fluid and Analysis method	No. of pts.	Significance	Area under the Curve (AUC) SS/SP
miR-21 miR-10b miR-200	↑ ↑ ↑	Teplyuk, 2012 [127]	CSF qRT-PCR	19 GBM 118 other non glioma – metastases, neurologic conditions	**miR-21** Increased in patients with GBM, brain metastases from breast and lung cancer compared to healthy controls Does not distinguish between primary glioma and brain metastases **miR-10b** Absent in controls Present in GBM, and brain metastases **miR-200** Not present in normal brain tissue Very low levels in glioma High levels in other solid cancers Its presence in CSF distinguishes between glioma and brain metastases *Quantification of* ***7 miRNA*** *in CSF distinguishes between GBM and brain metastasis*	N/A
miR-21	↑	Akers, 2013 [126]	CSF qRT-PCR	13 GBM 14 Non-cancer controls 24 GBM 5 Non-cancer controls	Distinguishes between GBM and non-oncologic controls	AUC = 0.91 SS = 87% SP = 93%
miR-21 miR-15b	↑ ↑	Baraniskin, 2012 [125]	CSF qRT-PCR	10 Glioma 10 Neurologic diseased patients 23 PCNSL 7 Brain metastases	**miR-21** Elevated in all glioma, PCNSL, metastases compared to non-neoplastic controls Lower CSF levels in glioma, compared to metastases and PCNSL **miR-15b** Elevated CSF levels in glioma compared to control, metastasis and PCNSL Distinguishes between glioma and non-glioma patients	miR-21 = N/A miR-15b: Glioma vs healthy controls AUC = 0.96 SS = 90% SP = 94%
miR-451 miR-711 miR-935 miR-223 miR-125b	↑ ↑/↓ ↓ ↑ ↑	Drusco, 2015 [129]	CSF Nano-String qRT-PCR	9 Glioma 2 Ependimoma 4 Meningioma 4 Glioblastoma 3 Medulloblastoma 4 Lung cancer metastasis 5 BC^9^ metastasis 3 Lymhoma 14 Healthy Controls	Differential expression of aforementioned miRNAs Levels could distinguish between cancer patients and healthy controls	N/A
miR-21-5p miR-218-5p miR-193b-3p miR-331-3p miR-374a-5p miR-548c-3p miR-520f-3p miR-27b-3p miR-130b-3p	↑ ↑ ↑ ↑ ↑ ↓ ↓ ↓ ↓	Akers, 2017 [128]	CSF TaqMan OpenArray Real-Time PCR System	*Cohort 4 – Cisternal CSF* 10 GBM 12 Controls *Cohort 5 – Lumbar CSF* 18 GBM 20 Controls	MiRNA signature distinguishes GBM from healthy controls in cisternal and lumbar CSF specimens Lumbar CSF has low sensitivity	Cohort 4 – Cisternal CSF AUC = 0.75 (95% CI 0.53, 0.97) SS = 80%; SP = 67% Cohort 5 – Lumbar CSF AUC = 0.83 (95% CI: 0.69, 0.96). SS = 28%; SP = 95%.

^1^*HGG* High grade glioma, ^2^*LGG* Low-grade glioma, ^3^*PCNSL* Primary central nervous system lymphoma, ^4^*GBM* Glioblastoma, ^5^*KPS* Karnofsky Performance Scale, ^6^*OS* Overall Survival, ^7^*PFS* Progression free survival, ^8^*DLGG* diffuse large grade glioma, *BC* breast cancer

Based on an exhaustive research of miRNA databases, scientific papers on microarray datasets and existing commercial PCR arrays, Toraih et al. propose an 84 miRNA panel to diagnose glioblastoma. Interestingly, the authors report a relatively modest overlap in both microarray datasets, as well as available ready-made miRNA panels. However, in the latter case, only 2 out of 4 miRNA panels (Qiagen, Exiqon) are brain tumor specific, while the remaining 2 – one screens for all types of cancer (GeneCopoeia) or is “customer-made array” (Life Technology – Thermo Fisher Scientific), this accounting for the observed heterogeneity [130]. Nevertheless, this initiative is promising, specialized diagnostic panels representing a step forward from scientific research to clinical practice.

Altogether these data show that miRNA have the potential to be the future biomarker for brain tumors that could solve crucial clinical problems: screen patients at risk for brain tumors, follow-up patients after surgery to monitor recurrence or even stratify patients in different risk groups.

By analyzing the data on miRNA biomarkers for brain tumors it is easy to observe that multiple problems exist. Firstly, some of the proposed miRNAs are not specific for brain tumors. For example, miR-21, miR-29, miR-125b, are documented to be found in other types of cancers [106, 111, 127]. Secondly, as mentioned, contradictory findings regarding miR-128 in glioma have been reported, found to be upregulated in one study [120], while being downregulated in others [100, 121].

Unfortunately, research is held back by the vast heterogeneity between studies, which makes it almost impossible to compare data between study groups and to summate the data in order to assess the value of miRNAs as biomarkers. In our view, this heterogeneity is also an important limitation of any attempt to perform a meta-analysis on this topic. The elements of heterogeneity are multiple and need to be outlined. Firstly, the study populations are from different ethnical groups. Differences in race specific miRNA expression have been already proven in hypertension, breast and prostate cancers [131–133]. This ethnical heterogeneity may also influence miRNA expression in brain cancers.

Secondly, the selection of body fluids varies throughout the studies. Even in blood derived products, studies report either using serum, plasma or blood cells, while studies focusing on CSF, extract it from lumbar or cisternal origin, this also accounting for heterogeneity. More than that, as Schwarzenbach et al. outline, miRNA expression levels can be influenced by various factors: starting with circadian rhythms, up to sample preservation, processing time, coagulation prevention and the level of hemolysis [134].

Thirdly, the RNA extraction techniques differ from study to study which is the case in our reviewed studies, where multiple extraction techniques have been employed. Kopkova et al. show how different RNA extraction kits and their usage can significantly influence expression results, advocating for the need of standardization [135].

Fourthly, the RNA detection method throughout studies is variable. A wide range of techniques have been employed (Nanostring, Solexa, TaqMan Openarray, Next Generation Sequencing), usually for initial screening, afterwards, selected miRNA expression levels being confirmed through quantitative RT-PCR. Again, Kopkova et al. suggest a significant expression variability, especially in screening techniques. Finally, there is great variability in qRT-PCR miRNA quantification in the presented studies, most of them using a relative quantification, but different molecules for normalization. Schwarzenbach et al. review how different normalizers can lead to significantly different quantifications of expression levels [134]. All these factors contribute to heterogeneous results in miRNA research.

We can envision different methods to improve the diagnostic power of miRNAs in brain tumors. Firstly, a strategy to expand the already existing miRNA panels as diagnostic tools is the use of the network theory. Each miRNA regulates tens to hundreds of mRNAs [136] and the intracellular mobility mechanisms of miRNAs suggests that this class of molecules are part of complex regulatory networks [137]. By using the expression of multiple miRNAs, it is possible to build miRNA networks, which contain not only data regarding the level of the miRNAs, but also characterize the relationship between miRNAs [138]. In various cancers, it was shown that compared to the normal status, the miRNA network becomes disconnected and fragmented [139].

Secondly, by adding other molecules with diagnostic potential to the miRNA panels, we could increase diagnostic accuracy. Circulating tumor DNA (ctDNA) has proven to be relatively abundant in the serum of patients with several human cancers, although in brain cancers the detection rate is lower [140]. Still, in this patient category ctDNA can be found more in CSF, where tumor-specific mutations can be detected, or even sequenced for mutation detection [140–142]. Research on lncRNAs also reported positive results regarding their use as biomarkers for brain tumors [143]. Even the role of circular RNAs, which are intertwined with miRNAs by acting as sponges, has been studied in glioma, and their implications in pathogenesis, progression, associations with pathological grade and prognosis have been reported, their potential use as biomarkers cannot be excluded [144, 145].

Thirdly, by having a clear picture of the miRNA bio-dynamics, understanding the mechanism through which miRNAs travel in blood or in the CSF could also improve the diagnostic method. A 2015 review by Witwer highlights many pitfalls in the common understanding of miRNA dynamics. Also, he underlines the role of cancer specific extracellular vesicles, and how analysis of surface lipids and proteins (e.g. EpCAM) of these vesicles could predict the origin and maybe even the destination of the vesicle and of its cargo, rendering better specificity in cancer diagnosis [146]. In our opinion, the merging of both EV surface proteins and miRNA contents and rendering of diagnostic trees may increase the diagnostic power of miRNAs in brain tumors.

## Conclusion

Despite tremendous efforts to develop new diagnostic and therapeutic tools to improve the survival in glioblastoma patients, minimal advances have been made. These efforts underline that a paradigm shift is necessary, a transition from protein based diagnostic biomarkers and therapies to RNA based ones.

Because of the proven roles played by miRNAs in gliomagenesis and of their capacity to pass from the CNS tissue into blood or CSF, we propose miRNAs as ideal diagnostic and prognostic biomarkers. In order to achieve this desiderate and confirm the potential of miRNAs a standardization of future studies is necessary: (a) use of similar biofluids for diagnostic; (b) use of similar RNA extraction methods; (c) use of similar normalization methods. Additionally, we consider that the specificity and sensitivity of diagnostic tests can be increased by using miRNA diagnostic trees or miRNA networks.

Moreover, miRNAs represent a possible new therapy for glioblastoma. Because of their wide mechanism of action, miRNAs are an ideal treatment for an extremely heterogeneous tumor type. In vivo therapy data shows that miRNAs can reactivate the immune system [69] or attenuate drug resistance [87] – two of the limitations of current therapies. One of the most important restrictions of this unmet medical need is the delivery of RNA therapeutics into the CNS, over the BBB. In recent years novel carriers were developed and synthesized which could overcome this limitation, and because of their structure and small molecular weight, miRNAs are the ideal loading of these delivery mechanisms.

## Abbreviations

AMOs: Antisense oligonucleotides
AQP11: Aquaporin-11
BBB: Blood-brain barrier
BCL2: B-cell lymphoma 2
CED: Convection-enhanced delivery
circRNAs: Circular RNAs
CNS: Central nervous system
CSF: Cerebrospinal fluid
ctDNA: Circulating tumor DNA
CVOs: Circumventricular organs
Evs: Extracellular vesicles
GM-CSF: Granulocyte-macrophage colony stimulating factor
HGG: High grade gliomas
ICH: Intracerebral hemorrhage
IDH: Isocitrate Dehydrogenase
INF-γ: Interferon-γ
KPS: Karnofsky Performance Scale
LGG: Low-grade gliomas
LNA: Locked nuclei acid
lncRNAs: Long non-coding RNAs
LPS: Lipopolysaccharide
MGMT: methylguanine-DNA methyltransferase
miRNAs: microRNAs
MRE: miRNA response element
ncRNA: Non-coding RNA
NPC: Neural precursor cells
OS: Overall survival
PCNSL: Primary Central Nervous System Lymphoma
PDPK1: 3-phosphoinositide-dependent protein kinase 1
RISC: RNA induce silencing complex
RNP: RNA nanoparticles
SFV-4: Semliki Forest virus-4
SOCS-1: Suppressor of cytokine signaling 1
S-TRAIL: Secreting type of tumor necrosis factor–related apoptosis inducing ligand
TGF-β: Transforming growth factor-β
Th1: T helper type 1 cells
TJ: Tight-junctions
TMZ: Temozolomide
TNF-α: Tumor necrosis factor-α
TRADD: TNF receptor associated death domain
TRADD: TNF receptor associated death domain
VE: Cadherin: vascular endothelial cadherin
VEGF: Vascular endothelial growth factor
ZO-1: Zonula occludens
